# Analysis of Midgut Microbial Diversity and Hemolymph Metabolomics in Silkworm (*Bombyx mori* L.) Varieties with Different Artificial Diet Feeding Habits

**DOI:** 10.3390/insects17060644

**Published:** 2026-06-18

**Authors:** Shengxiang Zhang, Yating Liu, Wenhui Song, Chunjiu Ren, Junwen Ai, Bing Han, Huiju Gao, Bing Wang

**Affiliations:** 1 State Forestry and Grassland Administration Key Laboratory of Silviculture in the Downstream Areas of the Yellow River, College of Forestry, Shandong Agricultural University, Tai’an 271018, China; zsx@sdau.edu.cn (S.Z.); 2 Yuelushan Laboratory, Changsha 410128, China; 3 Department of Microbiology, College of Life Sciences, Shandong Agricultural University, Tai’an 271018, China; 4 Institute of Cotton and Sericulture, Hunan Academy of Agriculture Science, Changsha 410127, China; 5 Liaoning Institute of Sericultural Science, Dandong 118000, China

**Keywords:** *Bombyx mori* L., midgut microbiota, metabolomics, L-valine, *Bacillus*

## Abstract

As important silkworm varieties reared on artificial diet, Youshi No. 1 (YS) and Guangshi No. 1 (GS) showed remarkable differences in physiological characteristics. GS had significantly better performance than YS in body weight, cocooning ability, food intake, feed utilization efficiency, and digestive enzyme activities. We further performed metabolomics and 16S rRNA high-throughput sequencing to analyze their metabolic profiles and midgut microbiota. More than 40 differential metabolites were screened out, and four metabolic pathways related to feeding divergence were determined via KEGG enrichment, among which L-valine was enriched in multiple pathways. Significant structural differences were also observed in midgut microbiota, and *Bacillus* was positively correlated with pantothenic acid and valine metabolism. These correlational results disclosed that differential metabolites and gut microbiota might underlie the phenotypic variations between the two varieties. Integrated analysis combined with functional verification experiments demonstrated that supplementation of 1% L-valine or specific *Bacillus* strains in an artificial diet was associated with the improvement of the growth performance, cocoon quality, and feed utilization efficiency of the YS silkworm variety.

## Simple Summary

As a model organism and an important economic insect, the feeding habit differences among various silkworm (*Bombyx mori* L.) varieties have long been a research focus. In this study, 16S rRNA high-throughput sequencing and metabolomic sequencing were employed to analyze the two artificial diet-reared silkworm varieties, Youshi No. 1 (YS) and Guangshi No. 1 (GS). The results revealed significant differences between their midgut microbiota and their metabolic profiles. L-valine was identified as a key metabolite significantly correlated with the growth and development of silkworms, and it showed a significant correlation with midgut bacteria *Bacillus*. Further verification experiments confirmed that exogenous supplementation of either L-valine or *Bacillus* significantly improved various physiological indices of YS silkworm reared on artificial diet.

## 1. Introduction

Silkworm is an important economic insect. With the promotion and application of artificial diets, a large number of varieties adapted to artificial diet feeding have been cultivated. These silkworm varieties show significant phenotypic differentiation in growth and development, silk quality traits, as well as feeding habits. Therefore, the formation mechanism among them has always been a hot topic in silkworm research. Currently, research in this field focuses mainly on analyzing the genetic and molecular basis of these phenotypic differences. For example, Jiang et al. revealed the differences in key genetic loci between the “anorexic” and “preferred” artificial diet varieties by comparative genomic analysis [1]; Ge et al. analyzed the differential regulatory mechanisms of the silk glands of hybrid silkworms based on genomic and proteomic data [2].

Compared with the genetic level, there is still a lack of systematic research on the gut microbiome structure among silkworm varieties with different adaptabilities and their interaction with host metabolism. Some studies have shown that there are significant differences in rumen microorganisms and metabolism among different sheep breeds, especially in amino acid and energy metabolism [3]. At the same time, some studies have pointed out that differences in gut microbiota and metabolites affect the meat quality characteristics of different pig breeds [4]. These findings provide important references for conducting research on the differences in gut microorganisms and metabolism among silkworm varieties.

The growth and development of the domestic silkworm were closely related to its metabolic activities. Amino acid metabolism, as a core biosynthetic and energy supply pathway, directly affects the synthesis of cocoon silk proteins, energy supply, and stress response in the domestic silkworm [5]. Metabolomic analysis of domestic silkworms reared on artificial diet revealed that amino acid metabolism was the most important pathway in the posterior silk gland (PSG) [6]. Studies on the feces of domestic silkworms reared on mulberry leaves and artificial diet found that most of the amino acids, carbohydrates, and lipids related to insect development and silk protein biosynthesis were enriched in domestic silkworms reared on mulberry leaves [7].

As the “second genome” of organisms, the gut microbiota was composed of various microorganisms such as bacteria and fungi colonizing the intestine. Research has shown that there is a close symbiotic relationship between the host and the gut microbial community, which has been confirmed in humans and various animals [8]. Silkworm is an important economic insect and model organism, and the functions and impacts of its gut microbiota are research hotspots. The intestine of the silkworm is composed of a large number of microorganisms, which play important roles in food digestion, host nutrition, immune response, and pathogen defense of the silkworm [9]. Many studies have been carried out on the gut microorganisms of the silkworm. Dong et al. compared silkworms fed with mulberry leaves and an artificial diet and found significant differences in the gut microbiota between them [10]; Chent et al. further explored the differences in gut microbiota composition between wild and domesticated silkworms [11]; Li et al. revealed the key role of symbiotic *Bacillus* in the growth, development, and metabolism of silkworms [12].

This study employed multi-omics approaches to analyze the physiological differences between two silkworm varieties reared on an artificial diet, Youshi No.1 (YS) and Guangshi No.1 (GS). It aimed to reveal the metabolic differences and the distinct compositions of gut microbiota among different silkworm varieties reared on an artificial diet. Validation experiments were designed to explore the key metabolites and key gut microbiota associated with different varieties of silkworms reared on an artificial diet, as well as the internal relationships between metabolites and microbiota, which provide a basis for the in-depth exploration of the mechanisms of physiological differences among different silkworm varieties reared on an artificial diet.

## 2. Materials and Methods

### 2.1. Tested Silkworms and Diet Preparation

The tested silkworm varieties were Youshi No. 1 and Guangshi No. 1 (abbreviated as YS and GS, artificial diet-reared varieties) hybridized and conserved in our laboratory. The silkworm larvae were reared at 27 ± 2 °C, with a humidity of 70–85%, and a 12 h light/12 h dark cycle. The diet was the M20 diet independently developed by our laboratory (composed of 30% mulberry leaf powder, 38% soybean meal powder, 29% corn flour, 0.08% vitamins B, 0.08% vitamins C, 1.1% inorganic salts), and the tested silkworms were reared with the diet throughout all instars.

### 2.2. Treatment

The tested silkworm varieties, YS and GS, were grouped at the start of the 5th instar. Each group had three replicates, with 70 silkworms in each group (3*n*, *n* = 70). The basic diet was the M20 diet.

### 2.3. Measurement of the Food Intake, Feed Utilization Efficiency, Body Weight, and Cocoon Quality

The daily body weight of silkworms in the YS group and GS group was determined at the 5th instar stage. On the third day of the 5th instar, the total weight of the silkworms in each group was weighed, and the daily amounts of the diet given were recorded. The same group received the same amount of diet on the same day. The excrement was removed from the silkworms every day, and the remaining diet and silkworm feces were kept for future use. After uniformly drying to a constant weight, we separated the remaining diet and silkworm feces, weighed them, and then calculated the food intake and the feed utilization efficiency. Each time the diet was given, an equal weight of diet for the determination of the diet’s moisture content was prepared. On the seventh day after the silkworms entered the cocooning frame, the cocoon shell weight, whole cocoon weight, and cocoon layer rate (cocoon shell weight/whole cocoon weight) of each group were investigated.

### 2.4. Determination of the Digestive Enzyme Activity and Gene Expression Level

Five silkworms were randomly selected from each group at the third day of the 5th instar from GS and YS groups, and each group was replicated 3 times. After 12 h of starvation, the silkworms were anesthetized on ice and placed on the dissection table, and their body surfaces were cut open with sterilized scissors. The intestinal fluid was collected into a 2 mL sterile EP tube and centrifuged at 5000 rpm for 10 min, and the supernatant was collected. α-Amylase and trypsin were measured using the test kit from Nanjing Jiancheng Bioengineering Institute, and the lipase was measured using the test kit from Point Biotechnology Co., Ltd. (Wuhan, China).

According to the above operation, the midgut tissues (3*n*, *n* = 5) of the GS and YS groups were taken out, washed 3 times with PBS buffer, placed in a 2 mL sterile centrifuge tube, and snap-frozen in liquid nitrogen, and then RNA was extracted using TransZol Up (TransGen Biotech, Beijing, China). cDNA was reverse-transcribed using the silkworm *RP49* gene (GeneID: 778453) as the housekeeping gene, and the expression levels of digestive enzyme genes were measured (Table S1).

### 2.5. Construction and Sequencing Analysis of Microbiota Sequencing Libraries

The midguts of 5th instar silkworms were obtained according to step 2.4. Nucleic acids were extracted using an OMEGA Soil DNA Kit. Specific primers 338F (5′-barcode+ ACTCCTACGGGAGGCAGCA-3′) and 806R (5′-GGACTACHVGGGTWTCTAAT-3′) were chosen for PCR amplification of the target region (338F and 806R are common universal primers for the 16S rRNA gene in microbial). The sequencing library was prepared by Shanghai Personal Biotechnology Co., Ltd. (Shanghai, China). Using a TruSeqNano DNALT Library PrepKit. Quality control was performed, followed by read denoising, paired-end merging and chimera filtering using the DADA2 pipeline via the QIIME2 command. After denoising of all libraries, the ASV characteristic sequences and ASV table were merged, and singleton ASVs were removed. Subsequent analysis was conducted through the Personal Cloud Analysis Platform (https://www.genescloud.cn, accessed on 2 September 2023).

### 2.6. Metabolomics Sample Preparation and Mass Spectrometry Analysis

The hemolymph of 3-day 5th instar silkworms of GS and YS were collected. For each replicate, 5 silkworms (*n* = 5) were tested. Two pairs of ventral legs of the silkworm were cut off with sterilized scissors, and the hemolymph was aspirated with a 100 μL pipette into a 1.5 mL sterile EP tube and centrifuged at 5000 rpm for 8 min. Then, the supernatant was obtained and sent to Shanghai Personal Biotechnology Co., Ltd. for library construction. With Proteo Wizard software (version 3.0), the data were converted into mzML format, and peak alignment was performed with XCMS software (version 4.3) to extract the peak area. Subsequent analysis was conducted through the Personal Cloud Analysis Platform.

### 2.7. Addition of Functional Bacteria and Culture Conditions

The three functional bacteria used in this study, namely *Bacillus velezensis* C4-5, *B. subtilis* C17, and *B. rugosus* WP-5, were obtained in the early stage of our laboratory. The strains were all cultured in LB medium (1% peptone, 0.5% yeast extract, 1% sodium chloride) at 37 °C for 12 h. The cells were obtained by centrifugation (4000 rmp, 10 min), washed three times with physiological saline, and then suspended at a concentration of 10^7^ CFU mL^−1^ for later use.

The newly molted 5th instar larvae of the YS variety were divided into the CK group, C4-5 group, WP-5 group, and C17 group, with three replicates in each group and 70 larvae in each group (3*n*, *n* = 70). The CK group was fed with the ordinary M20 diet evenly sprayed with normal saline every day, while the C4-5 group, WP-5 group, and C17 group were fed with the artificial diet evenly spread with bacterial liquid (based on the expected experimental effect, 1 mL of 10^7^ CFU mL^−1^ bacterial liquid was added to every 28 g of diet). The body weight of the third day 5th instar larvae and the quality of cocoons after mounting were determined.

### 2.8. L-Valine Supplementary Experiment

Newly molted 5th instar silkworms were divided into the GS-CK group, YS-CK group, and YS-1% group. The GS-CK group and YS-CK group were fed with the ordinary M20 artificial diet every day, while the YS-1% group was fed with the M20 diet uniformly mixed with 1% L-valine (*w*/*w*) every day.

### 2.9. Statistical Analysis

Data were expressed as the mean ± SEM. Statistical analysis was performed using SPSS v.20. *p* < 0.05 was considered statistically significant. Student’s *t*-test and ANOVA were used for comparisons among groups.

## 3. Results

### 3.1. Differences in Body Weight and Cocoon Quality Between Silkworm Varieties of YS and GS Reared on an Artificial Diet

**Figure 1:**
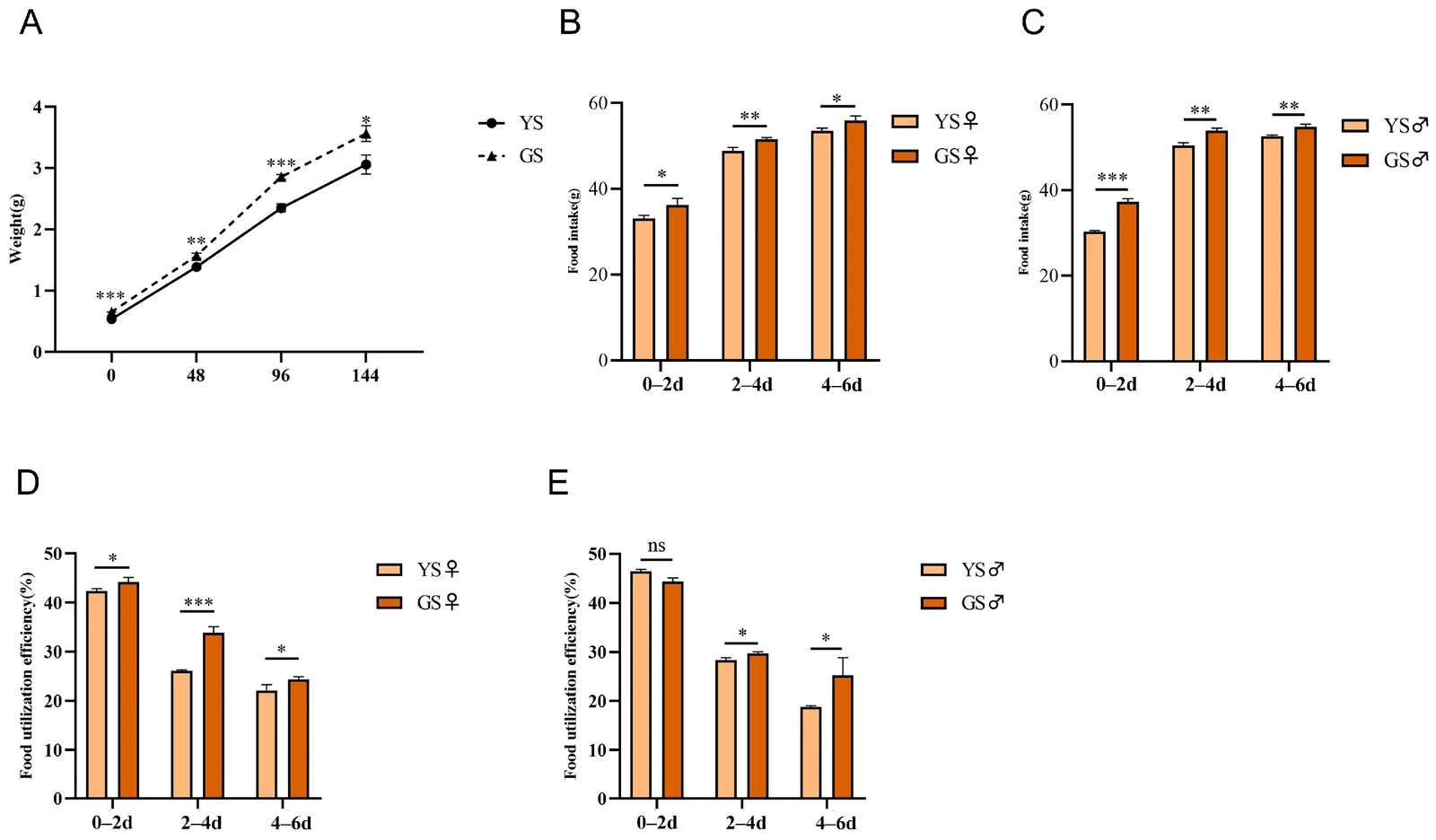
Physiological differences between YS and GS. (**A**) Changes in body weight and differences in body quality of YS and GS silkworms at the 5th instar; (**B**) food intake of female silkworms at the 5th instar stage of YS and GS; (**C**) food intake of male silkworms at the 5th instar stage of YS and GS; (**D**) differences in the feed utilization efficiency of female silkworms at the 5th instar age; (**E**) differences in the feed utilization efficiency of male silkworms at the 5th instar age. Data are presented as the mean ± SD. Differences in data were assessed by Student’s *t*-test. Statistical significance is indicated as follows: * *p* < 0.05; ** *p* < 0.01; *** *p* < 0.001; ns: not significant. The same below.

Both GS and YS had good adaptability to the artificial diet, but there were significant differences in their feeding characteristics. The body weights of YS and GS at 0 h, 48 h, 96 h, and 144 h of the 5th instar were measured. It was found that there were significant differences in the body weight of the two silkworm varieties during the 5th instar (Figure 1A). During the peak feeding period of the 5th instar, the average body weight of the GS group was 2.86 ± 0.04 g, while that of the YS group was only 2.35 ± 0.07 g. The difference between the two varieties was extremely significant, and the weight and volume of GS were significantly greater than those of YS (*p* < 0.001).

The results also showed that the food intake of GS was significantly higher than that of YS (Figure 1B,C). By investigating the feed utilization efficiency of silkworms, it was found that the feed utilization efficiency of female GS silkworms was significantly higher than that of female YS silkworms during 0–2 days of the 5th instar. The difference in the feed utilization efficiency between the two silkworm varieties was even more significant at 2–4 days. When approaching the mature stage at 4–6 days, the feed utilization efficiency of GS silkworms was still higher than that of YS silkworms (Figure 1D,E). This indicated that the significant difference in the food intake of artificial diet-reared silkworm varieties significantly affected the growth, development, and cocoon spinning ability of silkworms, while GS showed a higher feed utilization rate.

The total cocoon weight, cocoon shell weight, and cocoon layer rate of the GS group were all greater than those of the YS group (Table 1). Among them, the total cocoon weights of female and male silkworm pupa in the YS group were 1.74 ± 0.03 g and 1.46 ± 0.02 g, respectively, and the cocoon shell weights were 0.28 ± 0.01 g and 0.25 ± 0.01 g, respectively. The total cocoon weights of female and male silkworm pupa in the GS group were 2.15 ± 0.04 g and 1.77 ± 0.07 g, respectively, and the cocoon shell weights were 0.38 ± 0.01 g and 0.37 ± 0.02 g, respectively. The cocoon layer rates of female and male silkworm pupa in the YS group were 16.31 ± 0.55% and 17.13 ± 0.38%, respectively, and those of female and male silkworm pupa in the GS group were 17.93 ± 0.46% and 20.77 ± 0.15%, respectively.

### 3.2. Differences on Digestive Enzyme Activity and Gene Expression Levels Between Silkworm Varieties of YS and GS Reared on Artificial Diets

**Figure 2:**
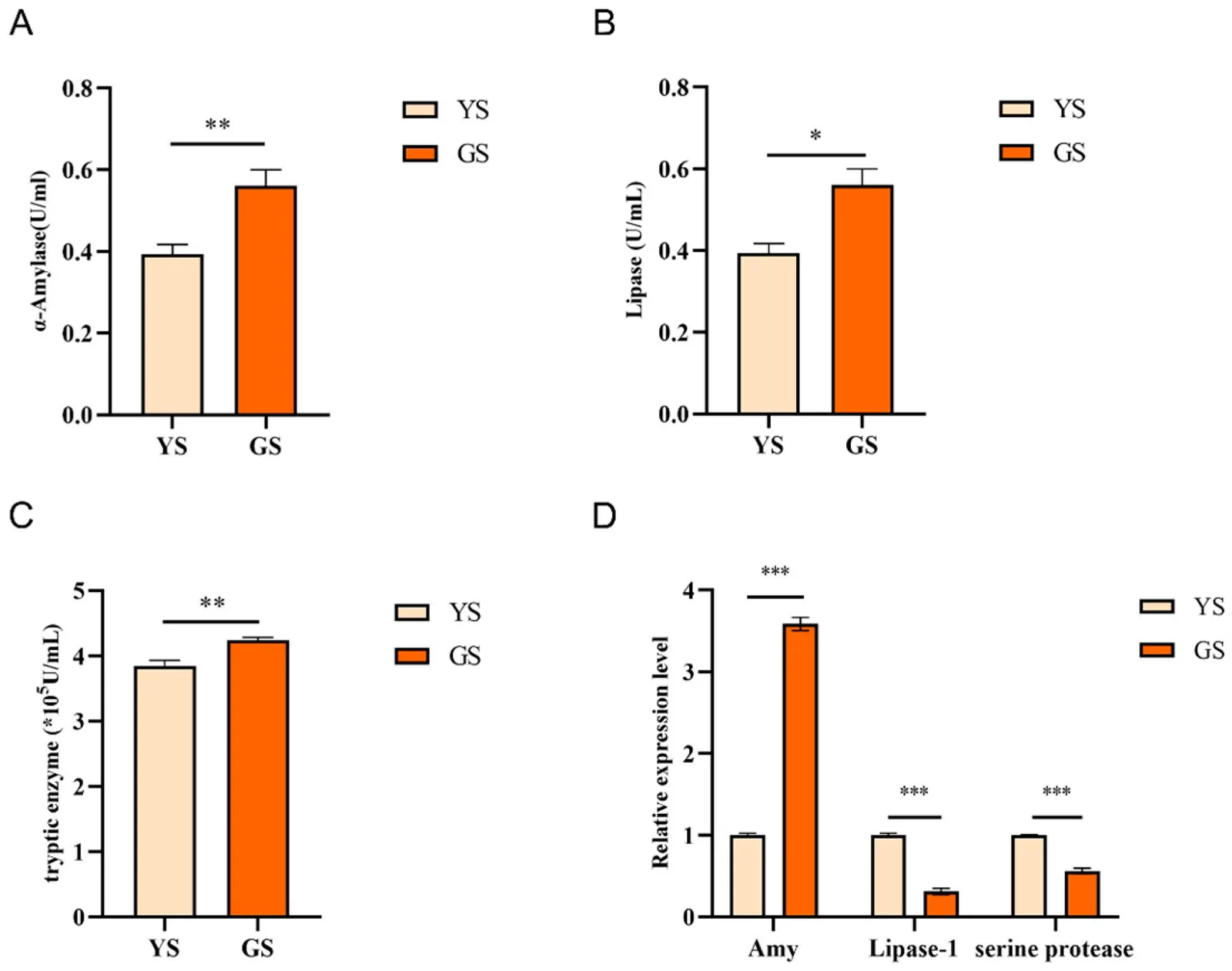
Differences in digestive enzyme activities and related gene expressions between YS and GS. (**A**) α-Amylase activity; (**B**) lipase activity; (**C**) trypsin activity; (**D**) relative expression of digestive enzyme genes. Differences in data were assessed by Student’s *t*-test. Statistical significance is indicated as follows: * *p* < 0.05; ** *p* < 0.01; *** *p* < 0.001.

The determination results of digestive enzyme activities showed that the activities of amylase, trypsin, and lipase in GS were significantly higher than those in YS (Figure 2A–C). It was speculated that the increase in the activities of digestive enzymes promoted the growth and development of silkworms. At the same time, the RT-qPCR results of digestive enzyme gene expression showed that the expression level of the α–amylase gene in the GS group was extremely significantly higher than that in the YS group, which was 3.58 times that of the YS group, consistent with the trend of enzyme activity differences. However, the expression levels of the lipase and serine protease genes in the YS group were significantly higher than those in the GS group (Figure 2D).

### 3.3. Differences in the Midgut Microbiology Composition Between YS and GS Silkworm Varieties Reared on Artificial Diets

**Figure 3:**
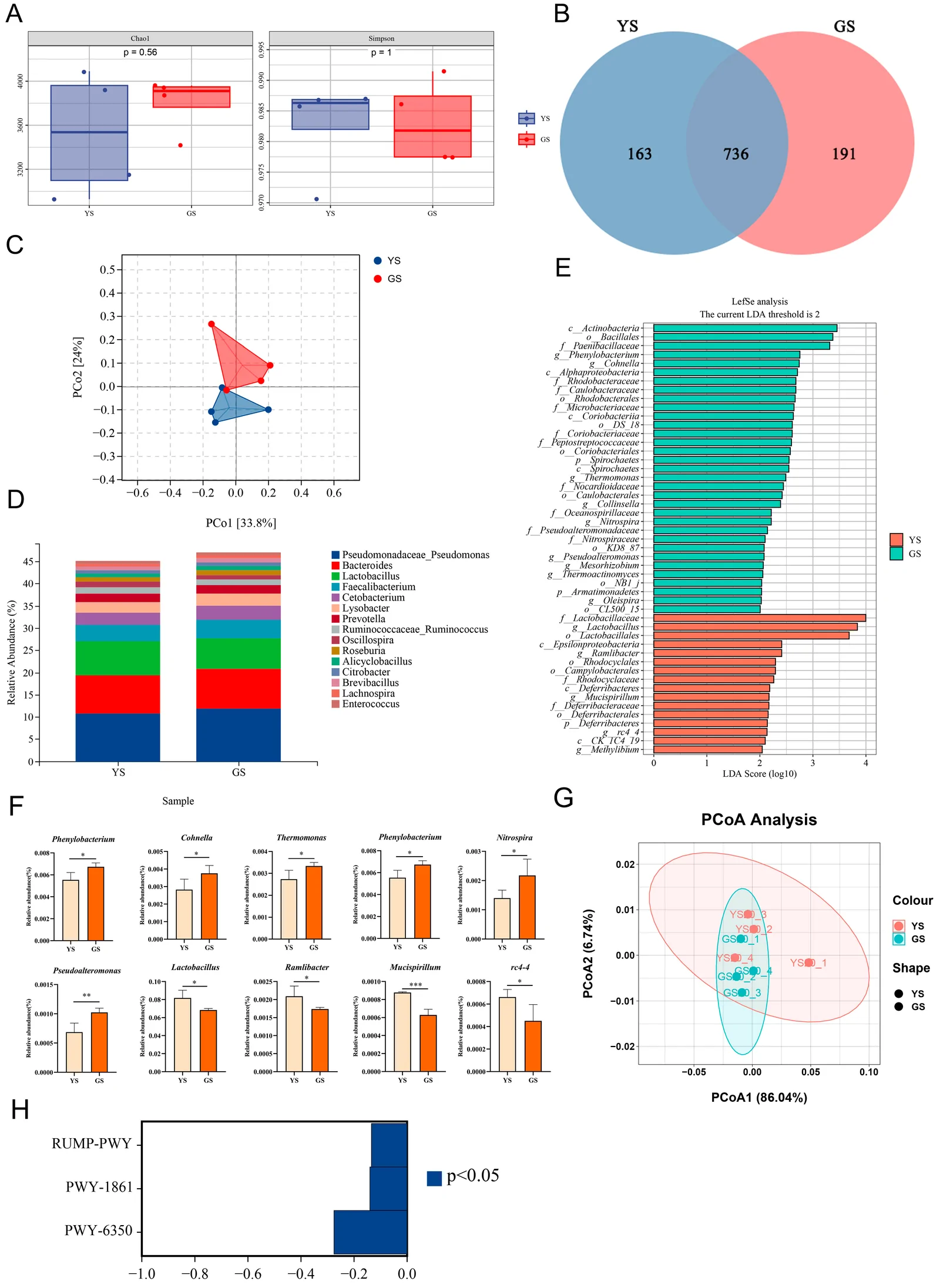
Structure and composition of the midgut bacterial flora in silkworm. (**A**) α diversity analysis of the differences between the two groups; (**B**) Venn diagram at the genus level of GS and YS; (**C**) PCoA of the differences between the two groups; (**D**) relative abundance of microbiology at the genus level in the midgut of silkworm; (**E**) analysis of the LEfSe results of the most significant differential biomarkers based on the LDA score (log 10); (**F**) equal abundance analysis of differential bacterial genera, Differences in data were assessed by Student’s *t*-test. Statistical significance is indicated as follows: * *p* < 0.05; ** *p* < 0.01; *** *p* < 0.001; (**H**) functional unit PCoA; (**G**) differential pathways in metabolic analysis (*p* < 0.05).

In order to explore the differences in gut microbiology between the GS and YS varieties, we studied their gut microbiology through 16S high-throughput sequencing technology. The results showed that there was no significant difference in the α-diversity of the microbiota between GS and YS (Figure 3A). The Venn diagram showed that there were 736 genera in common between the two group, 191 genera were unique to GS, and 163 genera were unique to YS (Figure 3B). The PCoA plot showed a clear separation of the GS and YS groups, indicating a significant difference in the midgut microbiota structure between the two varieties (Figure 3C). Further analysis showed that the dominant genera in the bacterial flora diversity at the genus level in GS and YS were *Pseudomonadaceae_Pseudomonas*, *Bacteroides*, *Lactobacillus*, and *Faecalibacterium* (Figure 3D). Compared with the YS group, the GS group increased the abundance of *Pseudomonas* in the midgut of silkworms and decreased the abundance of *Lactobacillus*. The LEfSe bar chart showed that there were significant differences in the abundance of bacterial flora in the midgut samples of the two groups of silkworms. The significantly different bacterial flora in GS were *Phenylobacterium*, *Thermomonas*, *Nitrospira*, *Cohnella*, *Collinsella*, and *Pseudoalteromonas*. The significantly different genera in YS were *Lactobacillus*, *Ramlibacter*, *rc4-4*, and *Mucispitillum* (Figure 3E). The analysis results of the relative abundance of differential flora supported the differences above (*p* < 0.05, Figure 3F).

We used PICRUSt2 to analyze the differences in metabolic pathways between YS and GS groups. The results of functional unit PCoA revealed clear separation and significant differences between the two groups (Figure 3G). Differential analysis screened out three metabolic pathways with significant differences (*p* < 0.05). The three pathways, namely RUMP-PWY, PWY-1861, and PWY-6350, were markedly downregulated in the YS group (Figure 3H). This indicated that the YS group reduced potential for xenobiotic metabolism and detoxification, as well as the metabolic activity of the microbiota.

### 3.4. Differences in Metabolic Pathways Between YS and GS Silkworm Varieties Reared on an Artificial Diet

**Figure 4:**
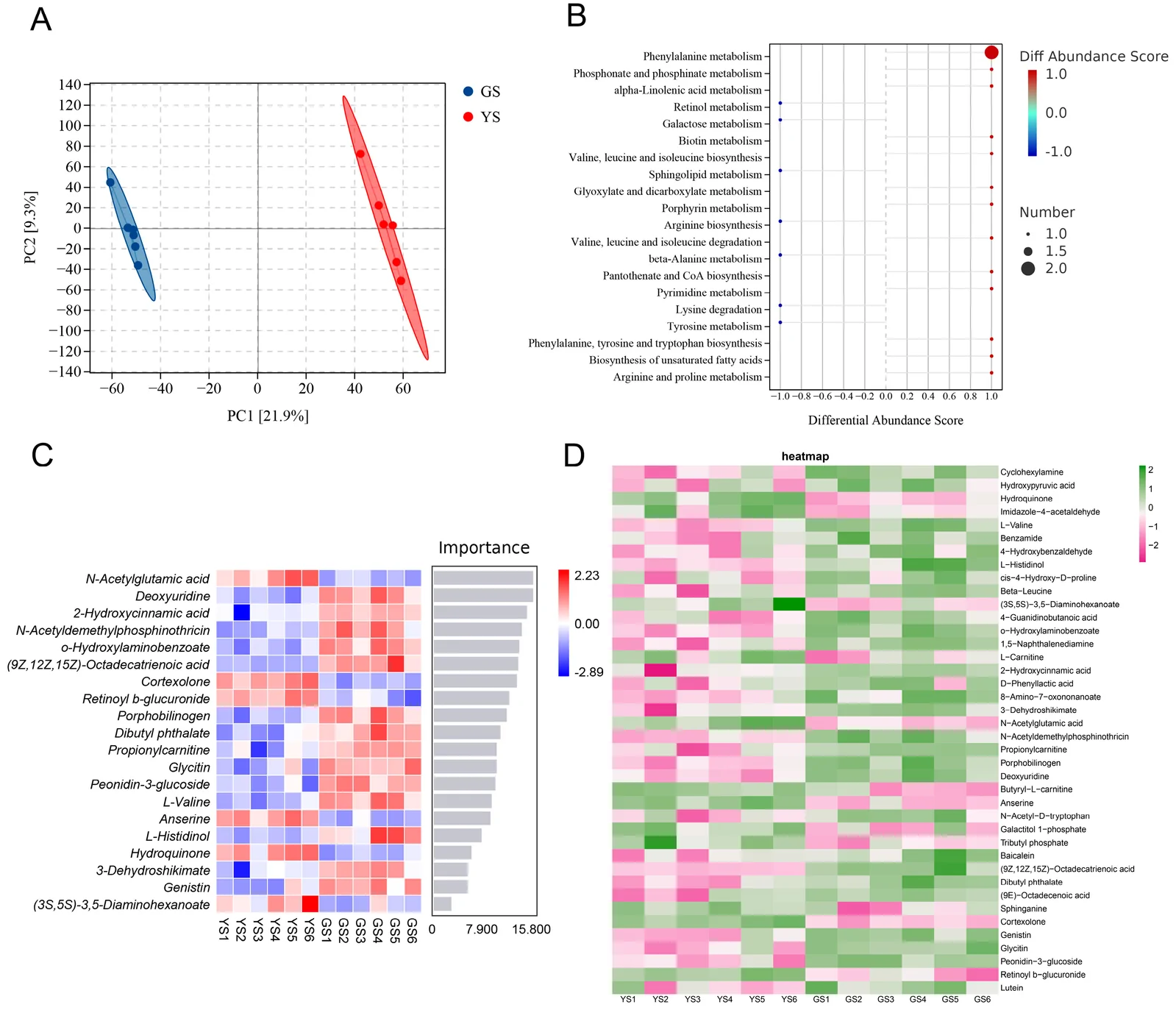
Non-targeted metabolomics of silkworm hemolymph and their combined analysis. (**A**) PLS-DA of the differences between the two groups; (**B**) top 20 differentially expressed metabolites ranked by abundance; (**C**) heatmap of differential metabolites; (**D**) random forest diagram of differential metabolites.

OPLS-DA showed that the samples of the YS and GS treatment groups were significantly separated, and there were obvious metabolic differences (Figure 4A, R^2^ = 0.97, Q2 = 0.21). The top 20 differential metabolic pathways showed that the significantly different metabolites were enriched in metabolic pathways such as phenylalanine metabolism, α-linolenic acid metabolism, biotin synthesis, and biosynthesis of unsaturated fatty acids (Figure 4B). Untargeted metabolomics analysis identified 40 differential metabolites. KEGG enrichment analysis revealed the pathways involved. Among them, there were 28 upregulated metabolites and 12 downregulated metabolites in the GS group compared with the YS group (Figure 4C). Compared with YS, the most abundant metabolites in the hemolymph of GS were Cyclohexylamine, L-Valine, L-Histidinol, and L-Glycitin. While compared with GS, YS had abundant Hydroquinon, N-Acetylglutamic acid, and Anserine in its body (Figure 4D). L-valine was enriched in multiple differential metabolic pathways, including the biosynthesis of L-valine, leucine, and isoleucine, degradation of L-valine, leucine, and isoleucine, biosynthesis of pantothenic acid and coenzyme A, ABC transporters, and biosynthesis of aminoacyl-tRNA (Table 2). Therefore, we speculated that L-valine was of great significance in promoting the growth and development of silkworms and increasing silk production.

### 3.5. Bacillus Showed a Positive Correlation with Valine Metabolism

**Figure 5:**
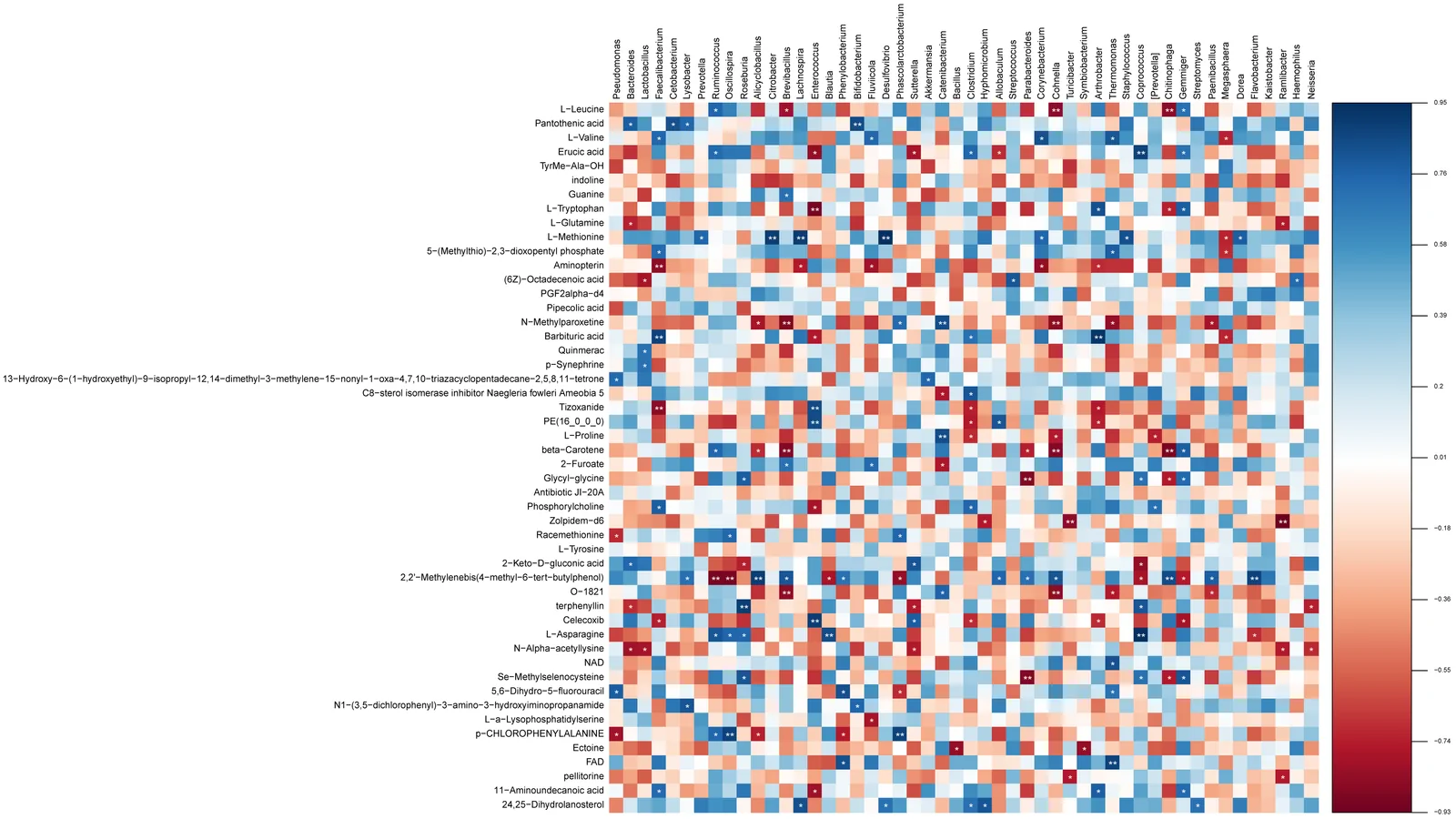
Heatmap of the correlation between differential metabolites and differential bacteria.* *p* < 0.05; ** *p* < 0.01.

The correlation matrix between differential metabolites and differential flora was calculated by the Spearman method (Figure 5). It was found that pantothenic acid had a significant positive correlation with 25 differential bacterial flora. Among them, it had a significant positive correlation with *Bacteroides*, *Cetobacterium*, *Lysobacter*, and *Bifidobacterium*. L-valine had a significant positive correlation with 26 differential flora and had a significant positive correlation with *Faecalibacterium*, *Fluviicola*, *Corynebacterium*, and *Thermomonas*. The silkworms of YS and GS, two artificial diet-bred varieties, were both rich in *Lactobacillus* at the genus level. It was positively correlated with pantothenic acid, indicating a correlation between lactic acid bacteria and pantothenic acid. The abundance of *Bacillus* in the GS group was significantly higher than that in the YS group, and it was positively correlated with pantothenic acid and L-valine. This indicated that the association among *Bacillus*, pantothenic acid, and L-valine involved the interaction between nutritional metabolisms.

### 3.6. Adding L-Valine to the Diet Promoted the Growth and Development of YS Silkworms

To further prove the effect of L-valine on the growth and development of silkworms, a preliminary experiment found that adding 1% L-valine to the artificial diet had the best effect on feeding the YS group, and the promotion effect was more obvious, especially in female silkworms. During the peak feeding period of the 5th instar, the body weight of YS-1% larvae was significantly higher than that of YS-CK, with an average weight of 1.88 ± 0.01 g per silkworm, which was significantly higher than that of the control group. The cocoon layer rate of the GS-CK group was 18.67 ± 0.02%. The total cocoon weight and cocoon shell weight of the larvae fed with 1% L-valine were the highest, which were 1.10 ± 0.04 g and 0.21 ± 0.05 g, respectively (Table 3). This indicated that feeding with L-valine significantly improved the growth and development of silkworms and the quality of cocoons, and the cocoon layer rate data of the YS-0.1% group was on par with that of the GS-CK group, which displayed excellent performance.

**Figure 6:**
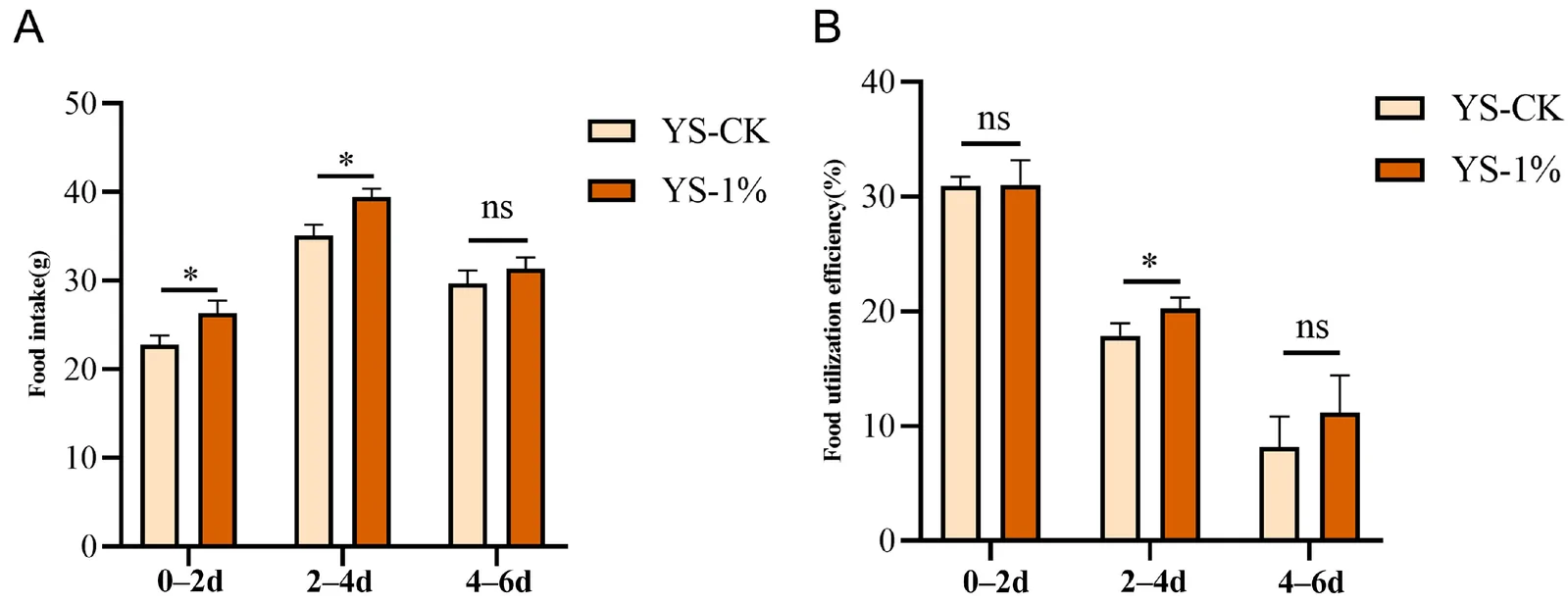
The feed utilization efficiency of silkworms fed with L-valine at the 5th instar. (**A**) The amount of food intake; (**B**) feed utilization efficiency. Differences in data were assessed by Student’s *t*-test. Statistical significance is indicated as follows: * *p* < 0.05; ns: not significant.

Indicators such as the daily food intake and feces weight of 5th instar silkworms were investigated every two days. The survey results showed that the food intake of YS-1% silkworms in the first four days of the 5th instar was significantly higher than that of the control group YS-CK (*p* < 0.05) (Figure 6A); during the 2–4 day of the 5th instar, the feed utilization efficiency of YS-1% was significantly higher than that of YS-CK (*p* < 0.05), which were 20.24 ± 0.99% and 17.89 ± 1.08%, respectively (Figure 6B). This indicated that after adding L-valine, the feed utilization efficiency of silkworms was improved, which had a positive effect on the growth and development of silkworms and the yield and quality of silk.

### 3.7. The Addition of Exogenous Bacillus Promoted the Growth and Development of Silkworms Reared on an Artificial Diet

**Figure 7:**
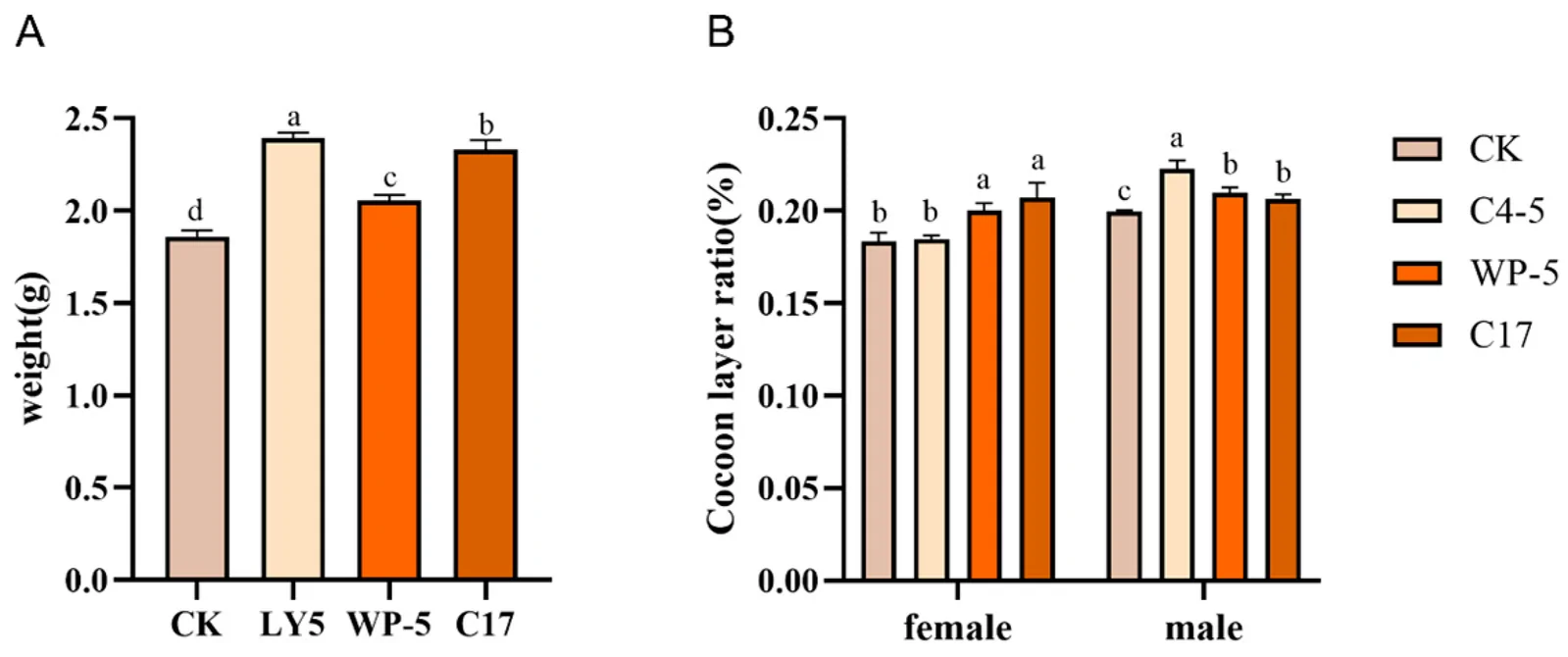
Effects of *Bacillus* supplementation on the body weight and cocoon quality of silkworms. (**A**) The body weight of the silkworm; (**B**) the cocoon layer ratio of the silkworm. Different letters indicate *p* < 0.05.

The experiment found that adding exogenous *Bacillus* to silkworms on the first day of the 5th instar effectively promoted the growth and development of silkworms, as shown in Figure 7. Different *Bacillus* species had different effects on silkworms. During the peak period on the third day of the 5th instar, the body weight of the CK group was 1.85 ± 0.04 g, that of the C4-5 feeding group was 2.38 ± 0.05 g, that of the C17 group was 2.31 ± 0.05 g, and that of the WP-5 group was 2.05 ± 0.03 g (Figure 7A). In terms of cocoon quality, on the seventh day of cocoon formation, the female cocoon layer rate of the CK group was 18.3 ± 0.4%, the male cocoon layer rate was 19.97 ± 0.05%; the female cocoon layer rate of the C4-5 feeding group was 18.4 ± 0.4%, the male cocoon layer rate was 22.25 ± 0.5%; the female cocoon layer rate of the WP-5 feeding group was 19.9 ± 0.4%, the male cocoon layer rate was 20.98 ± 0.2%; the female cocoon layer rate of the C17 feeding group was 20.7 ± 0.01%, and the male cocoon layer rate was 20.64 ± 0.3% (Figure 7B). After adding exogenous *Bacillus*, the body weight and cocoon layer rate of YS increased to varying degrees.

## 4. Discussion

### 4.1. Differences in Growth and Development Between Two Silkworm Varieties, YS and GS, Reared on Artificial Diets

Silkworm variety Guangshi No. 1 (GS) reared with an artificial diet had advantages such as wide food habit and fast growth characteristic. Youshi No. 1 (YS) had higher requirements for the mulberry leaf content in the artificial diet, and its growth situation was significantly different from that of GS, with relatively strong disease resistance. This study found that the body weight and cocoon-forming ability of YS were significantly lower than those of the GS group. At the same time, we found that the food intake and feed utilization efficiency of GS were significantly better than those of YS, and the activities of digestive enzymes were significantly higher than those of YS. Digestive enzymes play an important role in digestion and absorption during the larval stage of silkworms [13]. α-Amylase promotes the enzymatic hydrolysis of carbohydrates in the intestine [14]. Lipase decomposes dietary lipids into free fatty acids and glycerol [15]. Protease is highly expressed in the mid-gut and is crucial for protein digestion [16]. Therefore, we considered that the difference in digestive enzyme activities might be one of the important factors related to the growth differences between the two silkworm varieties, YS and GS.

### 4.2. There Were Differences in Multiple Metabolic Pathways Between the Two Artificial Diet-Reared Silkworm Varieties, YS and GS

In the study of hemolymph metabolism of two silkworm varieties, we found more than 40 differential metabolites. Compared with YS, the most abundant metabolites in the hemolymph of GS were cyclohexylamine, L-valine, L-histidinol, and glycine. And these three amino acids were simultaneously the marker differential substances of the GS variety. Amino acids were always considered as important substances restricting animal growth [17]. A series of studies have shown that the lack of branched-chain amino acids had a negative effect on fat deposition in mice [18,19], while exogenous addition of amino acids affected the expression of storage proteins through the amino acid–mTOR signaling pathway [20]. KEGG analysis in our study revealed that four metabolic pathways that affected the feeding habit differences were screened out from 20 significantly enriched metabolic pathways, namely phenylalanine metabolism, pantothenic acid and coenzyme A biosynthesis, ABC transporters, and aminoacyl-tRNA biosynthesis. Previous studies have shown that the lack of pantothenic acid-synthesizing bacteria in the intestine reduced the survival rate and reproductive ability of whiteflies [21], and Blow et al. found that aphids on a pantothenic acid-free diet grew poorly [22]. Therefore, we speculated that the synthesis of pantothenic acid and coenzyme A had an important impact on improving the growth, development, and feeding habits of silkworms. ABC transporters were a class of transmembrane proteins that participated in the absorption and transportation of nutrients across silkworm intestinal cells. Research has shown that if the function of ABC transporters is affected, it influences the absorption efficiency of nutrients in silkworms, which affect their feeding habits, growth, and development [23]. Enrichment analysis in our study found that valine was enriched in multiple differential metabolic pathways such as the biosynthesis of valine, leucine, and isoleucine, the degradation of valine, leucine and isoleucine, the biosynthesis of pantothenic acid and coenzyme A, ABC transporters, and aminoacyl-tRNA biosynthesis. Therefore, although genetic factors remain an important aspect to be considered, we speculate that the metabolite valine is likely closely associated with silkworm growth and development, and further mechanistic exploration is required in the follow-up research.

### 4.3. There Were Significant Differences in the Gut Microbiota Between the Two Artificial Diet-Reared Silkworm Varieties, YS and GS

The close correlation between the host and the gut microbiota is widespread in animals and humans. For silkworms, the structure and composition of the gut microbiota play an important role in their health and physiological state, involving functions such as food digestion, nutrient synthesis, resistance to pathogenic microorganism invasion, and immune regulation [24]. In this study, the midgut flora of different silkworm varieties, GS and YS, was analyzed by 16srDNA high-throughput sequencing. Our study found that, at the genus level, *Pseudomonades*, *Bacteroides*, and *Lactobacillus* were the common major dominant flora, accounting for nearly 30% of the total flora content. This finding is consistent with the dominant phylum level of silkworms, as pointed out in previous studies in which different diets affected the structural composition of the silkworm gut microbiota. Compared with silkworms fed an artificial diet, the dominant flora of silkworms fed mulberry leaves at the phylum level were Cyanobacteria, Firmicutes, and Proteobacteria, which was obviously related to changes in the diet structure [10].

This study found that varietal differences led to significant changes in the composition of the midgut bacterial flora. Compared with the YS group, the relative abundances of *Pseudomonades* and *Bacteroides* in the GS group increased, while the relative abundance of *Lactobacillus* decreased. It is worth noting that *Lactobacillus* and *Pseudomonades* have been confirmed to be closely related genera in silkworms reared on artificial diets [25], and *Pseudomonades* is considered to inhibit the reproduction of fungi. Previous studies have shown that silkworms with a lower abundance of *Pseudomonades* in the midgut are more susceptible to fungal diseases [11]. Some research has found that *Lactobacillus*, as a dominant genus, is present in both silkworms reared on artificial diets and those reared on mulberry leaves, while it is more abundant in silkworms reared on artificial diets [25]. In this study, *Lactobacillus*, as the dominant genus in the midgut flora of the two tested silkworm varieties, had a higher content in the YS group. However, the growth and cocoon quality indicators of YS variety silkworms reared on artificial diets were significantly lower than those of the GS group. We speculated that an excessive abundance of *Lactobacillus* was not directly conducive to the growth and development of silkworms reared on artificial diets. The possible reason for this adverse effect is that *Lactobacillus* might produce organic acids such as lactic acid, which significantly reduces the pH value of the silkworm gut. Since the gut environment of silkworms is alkaline, excessive lactic acid secretion would disrupt the pH balance of the silkworm gut, which is not conducive to the digestion and absorption of artificial diets and results in a delay in the growth and development of silkworms reared on artificial diets [26].

Combined microbiome and metabolome analysis in our study revealed that *Bifidobacterium* and lactic acid bacteria were positively correlated with pantothenic acid. In numerous health-oriented studies, *Bifidobacterium* has been widely recognized as a beneficial gut bacterium for host health [27]. However, verification experiments were difficult to perform due to its strictly anaerobic growth requirements, which demand high standards for anaerobic equipment and experimental operations. This study found a positive statistical correlation between *Bacillus* and the metabolic processes of pantothenic acid and L-valine. As a dominant bacterial genus in the silkworm gut, *Bacillus* plays a crucial role in the growth and development of silkworms. The direct effects of *Bacillus* on the growth and development of silkworms reared on artificial diets need further experimental verification.

### 4.4. Validation of the Gowth Pomoting Effects of L-Valine and Bacillus on Silkworm Variety YS Reared on an Artificial Diet

Based on the above findings, this experiment selected L-valine as the target differential metabolite and conducted feeding verification with L-valine and various *Bacillus* strains on silkworm YS reared on an artificial diet. The results showed that, for the YS group, larvae fed with 1% L-valine exhibited a significantly higher body mass, food intake, cocoon shell ratio, and feed utilization efficiency than the YS control group, with no significant difference compared with the GS control group. This result demonstrated that supplementation of L-valine in an artificial diet was associated with increased feed utilization efficiency and better growth, development, and cocoon performance of silkworms.

L-valine has been widely confirmed to play a crucial role in animal protein synthesis, feed utilization, energy metabolism, and maintenance of normal growth [28]. Studies have shown that exogenous L-valine supplementation promotes the growth, development, and cocoon quality of Liangguang II silkworms [29]. Yi et al. found that adding 0.24% L-valine to the diet of finishing pigs significantly increased their average daily weight gain [30]. These findings are consistent with the results of our L-valine feeding trial.

The feeding experiments with multiple *Bacillus* species in our verification experiment confirmed that *Bacillus* significantly promoted the growth, development, and cocoon spinning ability of silkworms. C4-5 significantly increased the body weight of silkworms, while C17 and WP-5 were more effective in improving the quality of silkworm cocoons. Some previous studies have shown that adding *Bacillus* to diet effectively increased the daily weight gain of infected pigs [31], and research has indicated that microbial metabolites and derivatives affect the organisms themselves [32,33]. For example, the metabolites of *Lactobacillus* affect the viability of colon cancer cells [34]. Therefore, we speculated that the metabolites of *Bacillus* might be closely correlated with the improved growth performance of silkworms.

## 5. Conclusions

In this study, GC-MS metabolomics and 16S rRNA gene high-throughput sequencing were integrated to analyze metabolic differences and intestinal microbial structural characteristics between the two silkworm varieties, YS and GS. The results revealed that valine was a key metabolite significantly correlated with silkworm growth and development, and it had a strong statistical correlation with bacterium *Bacillus*. Functional verification experiments further indicated that the exogenous addition of L-valine or *Bacillus* was associated with the improvement of various physiological indices of the YS silkworm reared on an artificial diet. Our data suggest that silkworm gut microbiota and their metabolites might be linked to multiple metabolic activities and intestinal digestive enzyme activities in the midgut, which were correlated with the differences in feeding performance, body weight, and cocoon quality among different artificial diet-reared silkworm varieties.

## Figures and Tables

**Table 1:** Investigation on cocoon quality of tested silkworm varieties of YS and GS.

		Youshi No. 1 (YS)	Guangshi No. 1 (GS)	*p* Value
Female	Whole cocoon weight	1.74 ± 0.03 g	2.15 ± 0.04 g	*** *p* < 0.001
	Cocoon shell weight	0.28 ± 0.01 g	0.38 ± 0.01 g	*** *p* < 0.001
	Cocoon shell ratio	16.31 ± 0.55%	17.93 ± 0.46%	* *p* < 0.05
Male	Whole cocoon weight	1.46 ± 0.02 g	1.77 ± 0.07	** *p* < 0.01
	Cocoon shell weight	0.25 ± 0.01 g	0.37 ± 0.02 g	*** *p* < 0.001
	Cocoon shell ratio	17.13 ± 0.38%	20.77 ± 0.15%	** *p* < 0.001

Data are presented as the mean ± SD. Differences in data were assessed by Student’s *t*-test. Statistical significance is indicated as follows: * *p* < 0.05; ** *p* < 0.01; *** *p* < 0.001.

**Table 2:** Up-regulated metabolites enriched in metabolic pathways.

Pathway	Up_Meta
Phenylalanine metabolism	2-Hydroxycinnamic acid, D-Phenyllactic acid
Phosphonate and phosphinate metabolism	N-Acetyldemethylphosphinothricin
alpha-Linolenic acid metabolism	(9Z,12Z,15Z)-Octadecatrienoic acid
Biotin metabolism	8-Amino-7-oxononanoate
Valine, leucine and isoleucine biosynthesis	L-Valine
Glyoxylate and dicarboxylate metabolism	Hydroxypyruvic acid
Porphyrin metabolism	Porphobilinogen
Valine, leucine and isoleucine degradation	L-Valine
Pantothenate and CoA biosynthesis	L-Valine
Pyrimidine metabolism	Deoxyuridine
Phenylalanine, tyrosine and tryptophan biosynthesis	3-Dehydroshikimate
Biosynthesis of unsaturated fatty acids	(9Z,12Z,15Z)-Octadecatrienoic acid
Arginine and proline metabolism	(9Z,12Z,15Z)-Octadecatrienoic acid
Phosphonate and phosphinate metabolism	4-Guanidinobutanoic acid
Histidine metabolism	L-Histidinol
Glycine, serine and threonine metabolism	Hydroxypyruvic acid
D-Amino acid metabolism	cis-4-Hydroxy-D-proline
ABC transporters	L-Valine, Deoxyuridine
Aminoacyl-tRNA biosynthesis	L-Valine

**Table 3:** Effects of L-valine supplementation on the body weight of 5th instar silkworms and cocoon quality.

Group	Weight (g)	Whole Cocoon Weight (g)	Cocoon Layer Weight (g)	Cocoon Layer Rate (%)
GS-CK	2.22 ± 0.02 ^a^	1.23 ± 0.07 ^a^	0.23 ± 0.01 ^a^	18.63 ± 0.02 ^ab^
YS-CK	1.83 ± 0.02 ^c^	1.08 ± 0.07 ^bc^	0.19 ± 0.09 ^c^	17.84 ± 0.04 ^c^
YS-1%	1.88 ± 0.01 ^b^	1.10 ± 0.04 ^b^	0.21 ± 0.05 ^b^	18.99 ± 0.06 ^a^

Data are presented as the mean ± SD. Differences in data were assessed by ANOVA. Values with different lowercase letters in the same column indicate significant differences at *p* < 0.05.

## Data Availability

The raw data in this study have been deposited in the NCBI BioProject under PRJNA1474233 (http://www.ncbi.nlm.nih.gov/bioproject/1474233, accessed on 3 June 2026).

## Supplementary Materials

The following supporting information can be downloaded at: https://www.mdpi.com/article/10.3390/insects17060644/s1, Table S1: RT qPCR primer sequences used in the research.
