# Correction: Zhang et al. Analysis of Midgut Microbial Diversity and Hemolymph Metabolomics in Silkworm (*Bombyx mori* L.) Varieties with Different Artificial Diet Feeding Habits. *Insects* 2026, *17*, 644

**DOI:** 10.3390/insects17080813

**Published:** 2026-08-05

**Authors:** Shengxiang Zhang, Yating Liu, Wenhui Song, Chunjiu Ren, Junwen Ai, Bing Han, Huiju Gao, Bing Wang

**Affiliations:** 1 State Forestry and Grassland Administration Key Laboratory of Silviculture in the Downstream Areas of the Yellow River, College of Forestry, Shandong Agricultural University, Tai’an 271018, China; zsx@sdau.edu.cn (S.Z.);; 2 Yuelushan Laboratory, Changsha 410128, China; 3 Department of Microbiology, College of Life Sciences, Shandong Agricultural University, Tai’an 271018, China; 4 Institute of Cotton and Sericulture, Hunan Academy of Agriculture Science, Changsha 410127, China; 5 Liaoning Institute of Sericultural Science, Dandong 118000, China

## Additional Affiliations

In the published publication [1], there was an error regarding the affiliations for Shengxiang Zhang and Junwen Ai. In addition to affiliations 1 and 3, the updated affiliations should include Yuelushan Laboratory, Changsha 410128, China.

## Missing Funding

In the original publication, the funder Yuelushan Laboratory Breeding Program was incorrectly translated as Yuelushan Laboratory Seed Industry Special Project. The authors state that the scientific conclusions are unaffected. This correction was approved by the Academic Editor. The original publication has also been updated.
