# Supplementary material for: Analysis of Midgut Microbial Diversity and Hemolymph Metabolomics in Silkworm (Bombyx mori L.) Varieties with Different Artificial Diet Feeding Habits
Source: Insects. 2026 Jun 18;17(6):644. doi: 10.3390/insects17060644 (PMC13300308; doi:10.3390/insects17060644)
Supplement: Supplementary file 1 [file insects-17-00644-s001.zip › insects-4305103-supplementary.pdf]

Table S1. RT qPCR primer sequences used in the research

gene	primer sequence
Amy-F	AATACACTCCACTTGCTGCCGTTAC
Amy-R	AATACACTCCACTTGCTGCCGTTAC
Lipase-F	TACGCCTCCACTGTTCGTCA
Lipase-R	TCCGCGCTTACCAAATACGG
serine protease-F	GCACTGACGAATGGCAGAACTTC
serine protease-R	CCTGGGCTCTCCTGGTCCTC
RP49-F	T CAATCGGATCGCTATGACA
RP49-R	ATGACGGGTCTTCTTGTTGG
